# Do improvements in outreach, clinical, and family and community-based services predict improvements in child survival? An analysis of serial cross-sectional national surveys

**DOI:** 10.1186/1471-2458-11-456

**Published:** 2011-06-09

**Authors:** Nancy Binkin, Mickey Chopra, Aline Simen-Kapeu, Dirk Westhof

**Affiliations:** 1 Health Section, Programme Division, UNICEF, 3 UN Plaza, New York, NY, USA; 2 UNICEF Country Office, Yerevan, Armenia; 3 D336 Defence Colony, Delhi, India

## Abstract

**Background:**

There are three main service delivery channels: clinical services, outreach, and family and community. To determine which delivery channels are associated with the greatest reductions in under-5 mortality rates (U5MR), we used data from sequential population-based surveys to examine the correlation between changes in coverage of clinical, outreach, and family and community services and in U5MR for 27 high-burden countries.

**Methods:**

Household survey data were abstracted from serial surveys in 27 countries. Average annual changes (AAC) between the most recent and penultimate survey were calculated for under-five mortality rates and for 22 variables in the domains of clinical, outreach, and family- and community-based services. For all 27 countries and a subset of 19 African countries, we conducted principal component analysis to reduce the variables into a few components in each domain and applied linear regression to assess the correlation between changes in the principal components and changes in under-five mortality rates after controlling for multiple potential confounding factors.

**Results:**

AAC in under 5-mortality varied from 6.6% in Nepal to -0.9% in Kenya, with six of the 19 African countries all experiencing less than a 1% decline in mortality. The strongest correlation with reductions in U5MR was observed for access to clinical services (all countries: p = 0.02, r^2 ^= 0.58; 19 African countries p < 0.001, r^2 ^= 0.67). For outreach activities, AAC U5MR was significantly correlated with antenatal care and family planning services, while AAC in immunization services showed no association. In the family- and community services domain, improvements in breastfeeding were associated with significant changes in mortality in the 30 countries but not in the African subset; while in the African countries, nutritional status improvements were associated with a significant decline in mortality.

**Conclusions:**

Our findings support the importance of increasing access to clinical services, certain outreach services and breastfeeding and, in Africa, of improving nutritional status. Integrated programs that emphasize these services may lead to substantial mortality declines.

## Background

A series of recent metanalyses have identified over 60 interventions that are effective in reducing mortality in children under five years of age [1]. Broadly speaking, they can be divided into three groups: i) interventions for direct treatment of illnesses ii) preventive interventions such as immunization and iii) health promotion/behavioral change interventions such as breastfeeding. Coverage remains low for many of the interventions, and unless it can be accelerated, it is unlikely that the Millennium Development Goal (MDG) 4 of a two-thirds reduction in under-five mortality from 1990 by 2015 will be met at the global level [2]. Efforts have thus been intensified by UNICEF and others to assist countries in identifying the most effective packages of interventions to address their leading causes of death within the context of the country's health care system and identify the bottlenecks that would need to be overcome to achieve high coverage.

Although in fragile countries or those with minimal infrastructure there may be a role for programs delivering a limited number of interventions [3], there has been increased emphasis in recent years on the provision of broader packages that share a common delivery mechanism and/or target population (e.g., the treatment of diarrhea, pneumonia, and malaria at community or facility level or the bundling of interventions that can be delivered through periodic outreach, such as vaccination, vitamin A, and bed net distribution) [4,5]. Whilst there is a large and growing evidence base regarding the composition and packaging of essential interventions, the evidence for which types of interventions should receive priority when considering marginal investments by governments and donors is unclear. A persistent concern has been that merely increasing expenditure on priority sets of interventions will not be sustainable without commensurate investments in health systems. The first step in combining a focus on critical interventions with investments in health systems is to identify the different ways in which interventions are delivered.

There are essentially three domains for the delivery of such packages, as outlined in the 2004 World Development Report [6]. The first domain is clinically-based services delivered at the individual level, which include not only treatment of such major childhood killers as diarrhea, pneumonia and malaria but also the availability of skilled attendants at delivery and emergency obstetrical care. The second consists of services that can be delivered primarily through episodic contact or outreach and include immunization, distribution of insecticide-treated bed nets for the prevention of malaria, distribution of vitamin A, and antenatal care. The final domain is community and family-oriented services that support self-care and includes activities such as information and social support for breastfeeding promotion and appropriate child nutrition. The three domains operate against a complex backdrop of the social, physical, economic, and cultural environment, which may influence the extent to which the interventions achieve their potential for change.

A recent study has demonstrated that countries which are making progress towards MDG4 have in common the scaling up of services such as family planning and immunization, which are delivered through episodic contact, while the countries making the most progress have also successfully scaled up their clinic-based services [7]. Among those working in maternal and child health, however, there has been a great deal of controversy regarding which domain represents the most appropriate and cost-effective means to reduce mortality in countries which have made poor progress. Proponents of community-based services have argued that strengthening this approach presents a much quicker and pragmatic approach than the longer and more expensive building of clinic-based services in settings with existing low levels of coverage and health system infrastructure [8].

Many countries have undergone rapid changes in the three service domains and also in contextual factors such as female literacy and wealth. However the extent to which changes in these domains have had an impact on under-five mortality rates is not known. In this paper, we use data from sequential Demographic and Health Surveys (DHS) from 27 countries to examine the relationship between progress in the three service domains and average annual change (AAC) in under-five mortality rates.

## Methods

### The countries

The analysis was limited to 27 countries in Africa, Asia, the Middle East and North Africa, and Latin America and the Caribbean that 1) were among the 68 so-called "Countdown" countries with the highest burden of maternal and child deaths [2] and 2) had two sequential surveys with data available and of reasonable quality on a wide range of indicators. These countries included Benin, Burkina Faso, Cameroon, Chad, Eritrea, Ethiopia, Ghana, Guinea, Kenya, Madagascar, Mali, Malawi, Mozambique, Niger, Rwanda, Senegal, Tanzania, Uganda, and Zambia in Africa, Cambodia, India, and Nepal, and the Philippines in Asia, Egypt and Morocco in the Middle East and North Africa, and Bolivia and Haiti in Latin America and the Caribbean.

### The database and variables

For each of the 27 countries, data were abstracted on the under-five mortality rate/1000 live births data from the most recent and penultimate DHS surveys. Data on 22 variables that individually correlated with under 5 mortality and were indicators of various types of coverage or of contextual factors were abstracted from the same the DHS country reports for each survey [9]. As shown in Table 1, outreach interventions included nine immunization and antenatal care variables as well as the proportion of family planning demand met. The seven clinical service-based interventions were related to delivery services such as skilled birth attendant and C-section coverage, although they also included treatment for diarrhea and care-seeking for pneumonia symptoms. Community and family services included feeding practices, lack of toilets, nutritional status and total fertility patterns. In addition, seven contextual variables known to be associated with overall mortality such as female literacy, water and sanitation, household goods, gross domestic product, and adult HIV prevalence were also included. All but gross domestic product (GDP) data and HIV prevalence were obtained from the DHS reports, while GDP was obtained from the World Bank [10], and HIV prevalence among adults 15-49 years of age was abstracted from UNAIDS country-specific projections [11]. In the case of GDP and HIV data, the information abstracted was for the same years in which the most recent and penultimate DHS surveys had been conducted. Under-five mortality rates were based on the values obtained from the openly available Child Mortality Estimates electronic database (CME info) [12].

**Table 1:** Categorization of variables

Outreach services	Clinical services	Community/family services	Contextual factors
BCG vaccination	Delivered by skilled birth attendant	Underweight	TV at home
DTP3 vaccination	Delivered in health facility	Stunting	Electricity at home
Measles vaccination	Delivered by a doctor	Wasting	Education of women 15-19 years
Maternal tetanus vaccination	Delivered by C-section	Exclusive breastfeeding for 6 months	Sanitation
Fully vaccinated	Sought care for ARI symptoms	Breastfeeding within an hour of birth	Water supply
Possesses vaccine card	Diarrhea episode treated with oral rehydration	Total fertility rate	Gross domestic product
At least 4 antenatal care visits	therapy or recommended home fluids	Availability of toilet	Adult HIV prevalence
At least 1 antenatal care visit			
Family planning needs satisfied among married women			

For each country, AAC in under-five mortality rates was calculated as follows:

***ln (x_1_/x_0_)/years***, where x_1 _was the value from the most recent survey, x_0 _from the penultimate survey, and years was the number of years between the two surveys. Similarly, the AAC was calculated for each of the service and contextual variables.

### Statistical analysis

The 22 indicators in the outreach, clinical and community services domains were reduced to a smaller number of elements using principal component analysis (PCA), a multivariate statistical technique used to reduce the number of variables in a data set into a smaller number of dimensions [13]. In this method, correlated variables are grouped together and PCA creates uncorrelated indices or components, whereby each component is a linear weighted combination of the initial variables. The weights for each principal component are given by the eigenvectors using the correlation matrix. Components from outreach, clinical and community services with eigenvalues > 1 were extracted on the basis of the scree plot, the percentage of variation explained in the total data and evaluation of the factor loading matrix after orthogonal (varimax) rotation [13]. The factor loadings, shown in Table 2, reflected the contribution and direction of correlation between each variable and the component.

**Table 2:** Results of the principal component analysis describing factor loadings for each component in outreach, clinical and community services in all countries (n = 27)

Variables (V)	Outreach services			Clinical services		Community/ family services	
	Component 1	Component 2	Component 3	Component 1	Component 2	Component 1	Component 2
Measles vaccination	**0.4246**	0.0208	0.0165				
DTP3 vaccination	**0.4393**	-0.0066	-0.0037				
BCG vaccination	**0.4217**	-0.0208	0.0291				
Fully vaccinated	**0.4431**	-0.0037	0.0164				
Tetanus	**0.4177**	-0.1288	-0.1382				
Vaccination card	**0.2770**	0.2731	0.0988				
At least four antenatal visits	-0.0359	**0.7027**	-0.1839				
At least one antenatal visit	0.0122	**0.6432**	0.1615				
Family planning services	-0.0033	-0.0201	**0.9539**				
Proportion (%)	0.52	0.21	0.12				
Cumulative variance (%)	0.52	0.73	0.85				
Attended by a doctor at delivery				**0.4326**	-0.1232		
Access to a skilled birth attendant				**0.5105**	0.0896		
Institutional delivery				**0.5610**	-0.0424		
C-section				**0.4775**	0.0560		
Treatment for pneumonia				0.0732	**0.6880**		
Treatment for diarrhea				- 0.0648	**0.7060**		
Proportion (%)				0.49	0.24		
Cumulative variance (%)				0.49	0.73		
Underweight						**0.5457**	0.2679
Stunting						**0.6132**	-0.1164
Wasting						**0.5872**	0.0303
Total fertility rate						**0.4759**	-0.1285
Exclusive breastfeeding for 6 months						-0.2402	**0.5521**
Breastfeeding within an hour of birth						0.2030	**0.4985**
Toilet						0.2658	**-0.3840**
Proportion (%)						0.32	0.29
Cumulative variance (%)						0.32	0.61

To provide an indication of the magnitude of the actual changes for each of the 9 identified components, the AAC for each indicator within the component was summed and divided by the number of indictors within the component for the 27 countries.

Linear regression was used to examine the relationships between the outcome variable (AAC in under-5 mortality between the most recent and penultimate DHS surveys) and explanatory variables (AAC in each outreach, clinical and family- and community-based component). This method estimates how much the value of the outcome changes when the values of the explanatory variable changes by one unit. In each linear regression model, we determined the robust standard error to control for heteroskedasticity. The amount of variance of the outcome explained by each predictor variable was estimated by R-square (r^2^). All estimates were adjusted for the confounding potential of contextual factors (displayed in table 1. They were also adjusted for the under-five mortality rates at baseline (the penultimate survey) as a means of increasing the comparability at the starting point of the 27 countries. All analyses were performed using Stata Version 11 (Stata Corp, TX, USA).

Because the influence of the three domains and contextual factors may vary in different settings, we performed parallel additional analyses for a subset of the 19 African countries in our study.

## Results

### Under-five mortality rates and average annual change (AAC)

As shown in Table 3, under-5 mortality in the penultimate surveys, conducted between 1992 and 2001, ranged from 60/1000 live births in the Philippines to 274/1000 in Niger. Values from the most recent surveys, which took place between 2002 and 2006, ranged from 37/1000 in the Philippines to 206/1000 in Chad. AAC in under 5-mortality also varied widely, from 6.6% in Nepal to -0.9% in Kenya, with six African countries all experiencing less than a 1% decline in mortality.

**Table 3:** Year of most recent survey, years since penultimate survey, mortality rates and average annual change in under-5 mortality

			Under 5 mortality rate/1000 live births		
Country	year of most recent survey	Years since last survey	penultimate survey	most recent survey	U5MR
Benin	2006	5.0	148	132	2.3%
Bolivia	2003	9.5	121	82	4.1%
Burkina Faso	2003	10.5	206	190	0.8%
Cambodia	2005	5.5	118	93	4.3%
Cameroon	2004	6.0	151	144	0.8%
Chad	2004	7.0	201	206	-0.4%
Egypt	2005	9.0	78	45	6.1%
Eritrea	2002	7.0	133	99	4.2%
Ethiopia	2005	5.0	167	138	3.8%
Ghana	2003	4.7	111	111	0.0%
Guinea	2005	6.0	204	175	2.6%
Haiti	2005	11.0	151	93	4.4%
India	2006	7.0	103	81	3.5%
Kenya	2003	5.0	111	116	-0.9%
Madagascar	2004	6.3	156	132	2.7%
Malawi	2004	4.0	192	152	5.8%
Mali	2006	5.5	229	201	2.4%
Morocco	2004	11.5	93	51	5.2%
Mozambique	2003	6.5	212	163	4.0%
Nepal	2006	5.0	96	69	6.6%
Niger	2006	8.0	274	204	3.7%
Philippines	2003	10.0	60	37	4.8%
Rwanda	2005	5.0	200	180	2.1%
Senegal	2005	8.0	148	125	2.2%
Tanzania	2004	8.0	156	131	2.2%
Uganda	2006	5.5	155	138	2.1%
Zambia	2002	5.5	178	178	0.0%

### Results of the principal component analysis

Results of the PCA for the 27 countries, including the factor loadings and the total variance explained, are shown in Table 2. The first PCA on outreach services reduced the nine variables to three components that explained 85% of the total variance: 1) immunization (BCG, measles, DTP3, maternal tetanus vaccination; fully vaccinated; and has vaccine card); 2) antenatal care (≥1 and ≥4 antenatal care visits) and 3) family planning (family planning need satisfied among married women). PCA was also used for item reduction of the six variables on clinical services to two components that explain 73% of the total variance: 1) access to services (physician as birth attendant, skilled birth attendant, institutional delivery, and Cesarean section); 2) seeking treatment (care-seeking for pneumonia and appropriate diarrhea treatment). The third PCA on community and family services reduced the seven variables to two components that explain 61% of the total variance: 1) nutritional status (underweight, stunting, and wasting) and 2) breastfeeding (breastfeeding within an hour of birth and exclusive breastfeeding for the first six months of life). The former component also included total fertility rate and the latter absence of toilet. We restricted the PCA analyses to the 19 African countries and found similar results (components) for outreach services (84% of variance explained), clinical services (69% of variance explained) and community and family services (67% of variance explained).

### Average annual change in outreach, clinical, and community and family components

The average of the AAC for each indicator in the nine domains is shown in Table 4. Of note are the wide variations in AAC for many of the components between countries, the limited magnitude of the changes in many of the countries, and the negative AAC for several components, especially in some of the African countries.

**Table 4:** AAC in components of outreach, clinical, and community and family services

	Outreach services			Clinical services		Community Services	
Country	Immunization	Antenatal care	Family planning	Access	Careseeking/ treatment	Nutrition	Breastfeeding
Benin	-0.1%	-0.1%	-2.2%	0.9%	-1.1%	-0.9%	4.1%
Bolivia	4.0%	5.1%	0.6%	3.5%	1.1%	4.6%	4.9%
Burkina Faso	1.1%	-0.1%	0.2%	-1.8%	4.2%	-1.3%	7.7%
Cambodia	8.1%	15.5%	6.8%	13.5%	1.8%	5.7%	17.1%
Cameroon	3.1%	1.6%	-1.0%	-0.8%	-0.6%	0.0%	2.9%
Chad	0.3%	4.9%	-15.1%	0.8%	-6.7%	0.3%	2.4%
Egypt	1.0%	7.3%	1.4%	7.7%	-1.8%	4.1%	-0.7%
Eritrea	7.4%	5.7%	0.3%	5.5%	2.9%	2.2%	2.3%
Ethiopia	7.1%	1.9%	9.8%	3.5%	-0.2%	1.6%	4.8%
Ghana	1.7%	1.6%	1.5%	-0.7%	4.8%	1.4%	8.6%
Guinea	1.2%	1.7%	6.3%	-0.1%	-1.1%	-1.9%	8.0%
Haiti	2.4%	2.9%	4.3%	3.3%	2.7%	1.3%	4.2%
India	1.7%	3.1%	1.1%	2.5%	0.3%	0.2%	3.9%
Kenya	-1.5%	-2.0%	0.0%	-3.2%	-4.8%	1.2%	-0.4%
Madagascar	3.5%	0.4%	3.4%	0.6%	1.2%	-1.8%	7.1%
Malawi	-0.9%	0.3%	2.1%	2.0%	5.4%	1.7%	1.9%
Mali	7.1%	3.4%	-1.0%	3.2%	-2.0%	-0.1%	5.3%
Morocco	-0.7%	7.3%	1.9%	7.9%	3.4%	-2.5%	-0.6%
Mozambique	4.6%	4.0%	5.7%	1.2%	3.8%	1.5%	0.0%
Nepal	6.1%	11.7%	2.4%	13.4%	3.7%	0.5%	2.0%
Niger	4.3%	2.7%	2.9%	3.1%	2.4%	1.8%	15.6%
Philippines	-0.2%	1.8%	1.9%	2.1%	1.9%	1.5%	2.8%
Rwanda	-0.6%	2.7%	2.9%	-0.1%	6.4%	2.6%	-0.2%
Senegal	2.5%	5.9%	0.0%	1.1%	1.6%	1.7%	7.3%
Tanzania	-0.8%	2.1%	3.3%	2.1%	-1.3%	4.3%	2.0%
Uganda	4.4%	1.2%	-1.3%	3.0%	0.9%	0.4%	-1.7%
Zambia	-2.1%	-0.2%	2.1%	-1.1%	-1.3%	-1.9%	4.2%

Values represent the average of the AAC for each indicator within the component.

### Correlation between AAC in outreach components and AAC in under-five mortality

The results of the linear regression analyses for all countries showed that an increase in antenatal (p = 0.006, r^2 ^= 0.38) and family planning (p = 0.02, r^2 ^= 0.32) services delivery through outreach or episodic activities was correlated with a decline in under-five mortality rates (Table 5). In the 19 African countries, changes in antenatal services was the only component that was correlated to a decrease in under-five mortality rates (p = 0.04, r^2 ^= 0.29) (Table 6). Overall, the analyses showed poor correlation for immunization services.

**Table 5:** Linear regression analysis examining the correlation between average annual change in under-5 mortality rates and average annual change in each component within outreach, clinical and community services in all countries (n = 27)

Component	Coefficient	95% Confidence interval	p value*	r^2^†
**Outreach services**				
Component 1: **Immunization**	0.0032	-0.0004, 0.0069	0.08	0.09
Component 2: **antenatal care**	0.0091	0.0029, 0.0152	**0.006**	0.38
Component 3: **family planning**	0.0090	0.0017, 0.0163	**0.018**	0.32
**Clinical services**				
Component 1: **access to services**	0.0091	0.0052, 0.0129	**0.018**	**0.58**
Component 2: **seeking treatment**	0.0074	0.0012, 0.0136	**0.021**	0.31
**Community/ family services**				
Component 1: **Nutritional issues**	0.0019	-0.0081, 0.0042	0.52	0.14
Component 2: **breastfeeding**	0.0087	0.0028, 0.0186	**0.006**	0.38

*p value: significance of the correlation between the AAC in each component in specific interventions and the AAC in under-5 mortality rate

† r^2^ is the proportion of variability in the AAC in under-5 mortality rate that can be explained by changes in the values of each component

Note: All analyses were adjusted for AAC in GDP, adult HIV prevalence, household TV and electricity, education of women 15-19, sanitation, after supply, and for under-5 mortality rate during the penultimate survey

**Table 6:** Linear regression analysis examining the correlation between average annual change in under-5 mortality rates and average annual change in each component within outreach, clinical and community services in African countries (n = 19)

Component	Coefficient	95% Confidence interval	p value*	r^2^†
**Outreach services**				
Component 1: **Immunization**	0.0025	-0.0030, 0.0081	0.33	0.10
Component 2: **antenatal care**	0.0111	0.0006, 0.0216	**0.039**	0.29
Component 3: **family planning**	0.0060	-0.0049, 0.0170	0.25	0.12
**Clinical services**				
Component 1: **access to services**	0.0118	0.0068, 0.0167	**0.000**	**0.67**
Component 2: **seeking treatment**	0.0064	-0.0017, 0.0146	0.116	0.19
**Community/ family services**				
Component 1: **Nutritional issues**	0.0110	0.030, 0.0190	**0.01**	0.40
Component 2: **breastfeeding**	0.0058	-0.0023, 0.0139	0.17	0.15

*p value: significance of the correlation between the AAC in each component in specific interventions and the AAC in under-5 mortality rate

† r^2^ is the proportion of variability in the AAC in under-5 mortality rate that can be explained by changes in the values of each component

Note: All analyses were adjusted for AAC in GDP, adult HIV prevalence, household TV and electricity, education of women 15-19, sanitation, after supply, and for under-5 mortality rate during the penultimate survey

### Correlation between AAC in clinical components and AAC in under-five mortality

As shown in Tables 5 and 6, change in access to clinical services for delivery demonstrated the strongest correlation with declines in under-five mortality for all countries (p = 0.02, r^2 ^= 0.58) and for the 19 African countries (p < 0.001, r^2 ^= 0.67). AAC in care seeking treatment for pneumonia and diarrhea was correlated with change in under-five mortality for all 27 countries, but the association was weaker and non-significant for the subset of African countries (Table 6).

### Correlation between AAC in community and family components and AAC in under-five mortality

A moderate correlation was noted for the breastfeeding component when the analyses included all countries (p = 0.006, r^2 ^= 0.38) (Table 5), but a weaker and non-significant association was observed for the 19 African countries. By contrast, for the 19 African countries (Table 6), an improvements in childhood nutritional status were significantly correlated to a decline in under-five mortality rates (p = 0.01, r^2 ^= 0.40), while the relationship was weaker and non-significant for the full set of 27 countries.

## Discussion

Our study demonstrated considerable variability in the AAC in under-five mortality among the 27 countries and that the strongest predictor of changes in under-five mortality rates was improvements in access to and coverage of clinical services. In addition, improvements in selected outreach interventions were also correlated with changes in mortality, but the effect was not as strong as for clinical services. For community and family services, we found that an improvement in breastfeeding practices/hygiene was significantly correlated with under-five mortality but that the effect was weaker and not statistically significant among the African countries. By contrast, childhood nutritional status was strongly correlated to a positive impact in under-five mortality rates only in the 19 African countries.

The importance of access to clinical services is not completely surprising as the major causes of child deaths such as pneumonia, diarrhea, malaria and various conditions of the neonatal period historically have required access to clinical services provided through health facilities. Our findings are also in keeping with those of Rohde *et al *[7], who demonstrated that the countries that had made the most progress as measured by average annual reduction of mortality were those which had higher coverage of comprehensive primary health care, which in their study was measured by a high prevalence of skilled attendants at birth. They, too, suggest that countries can achieve progress in child mortality even in the absence of improvements in contextual factors such as per capita income and HIV prevalence.

The observed correlation between the changes in antenatal care and family planning outreach services and mortality, though weaker than that observed for access to clinical services, suggests that improvements in access to these services can also have an important impact on mortality. With respect to immunization, some studies have reported a positive effect of immunization on child mortality [14-16], while others failed to demonstrate its effect on mortality [17-19]. Indeed, in our study, annual increases in immunization coverage were not correlated with changes in under-five mortality rates, although clearly it is essential to maintain or improve such coverage. Furthermore, the anticipated addition of pneumococcal and rotavirus vaccines to many national immunization programs may well boost the impact of improved immunization coverage on mortality.

With respect to community and family services, improved breastfeeding coverage and an increase in households with improved sanitary facilities also had an impact on under-5 mortality, although the impact was less in Africa than in the 27 countries as a whole. Indeed, exclusive breastfeeding for 6 months has been identified as having the greatest potential impact of any single intervention on under-5 mortality [20]. Regarding nutritional status, malnourished children are known to be at an increased risk of death [21-23], and it has been estimated that malnutrition contributes to about one-third of the child deaths that occur each year [24]. Our study demonstrated that an improvement in childhood nutritional status is associated with a decline in under-5 mortality rates, although the difference was significant only in the subgroup of African countries. These findings are compatible with previous studies [23,25,26]. It has been estimated that implementing interventions that improve child nutrition as well as provide clean water and sanitation to all children younger than 5 years would result in an estimated 31% reduction in child deaths in sub-Saharan Africa [25].

While these findings support further scaling up services in the outreach and community and family domains, they also would appear to underline the importance of additional investment in access to services. Prior efforts to scale up of clinical services have demonstrated major challenges in identifying and training adequate skilled staff to provide these services and in providing the facilities and logistics to ensure quality and continuity. In addition, the high costs of delivering such services have been a major inhibitory factor. Indeed, the overall costs of improving and sustaining a system capable of delivering rapid gains in health improvement are large, estimated at $36-45 billion annually above and beyond current expenditures, and the difficulty of raising such funds given current worldwide economic conditions, is substantial [27]. At the same time, however, our findings suggest that the benefits of such improvements may also be large.

One of the reasons behind the high costs of clinical services is the difficulty of providing care to those most in need, many of whom live in remote areas where access may be difficult. An encouraging development that may improve clinical coverage and also offers some excellent opportunities for synergy with outreach and family and community efforts is integrated community case management (iCCM) [28]. In iCCM, treatment for conditions such as pneumonia diarrhea, and malaria, which account for approximately 40% of under-5 mortality worldwide [29], is delivered at community level by specially trained community health workers. Such efforts also have the advantage of emphasizing family and community-based prevention and promotion and provide opportunities to promote better coverage of outreach services, resulting an excellent opportunity for synergy of those interventions which appear to have the greatest impact on reductions in mortality.

While other analyses have focused on the relationship between prevalence or coverage of risk factors and current mortality levels in study populations or at country level [[7]30], to our knowledge, this is the first analysis that examined the correlation between changes in individual and composite variables and changes in under-5 mortality at country level. We believe this approach provides a more dynamic way of examining how the scaling up of different types of interventions effect changes in under-5 mortality rates that is not provided by the simple correlation of existing coverage and mortality levels.

At the same time, however, these findings need to be interpreted cautiously. First, the results may not be generalizable because of the limited number of countries included in the analysis. We believe, however, that the countries included represent a wide range of experiences, both in terms of geography and initial levels of development. A second limitation is that consistent data on potentially important variables such as vitamin A administration and use of insecticide treated bed nets, which are highly effective in preventing malaria, were not available from the penultimate surveys. Furthermore, it is clear that many of the variables we included were markers rather than directly associated with child mortality as an outcome. For example, changes in Cesarean section, which as an individual variable produced one of the highest correlations with under-5 mortality change, was less strongly associated with reductions in the infant mortality, as might have been expected with a more direct effect; which strongly suggests that it is a marker of access to quality clinical services. A fourth limitation was that some of the variables that might be more plausibly linked to mortality such as treatment for acute respiratory infection could not be included because the two-week reference frame in the questionnaire results in prevalence values of < 5%, making calculation of average annual change problematic. A fifth limitation concerns the assumption that the effects of individual interventions and their interactions will be similar for our countries, which may not be true as suggested by the differences observed between the overall set of countries and the African countries. Finally, mortality data collected in surveys reflect events occurring in the population an average of 2.5 years previously. The mortality figures we used thus reflect mortality that occurred in the early 2000s, before many of the interventions had been scaled up, which may underestimate the influence that changes in coverage have had on mortality.

Our study looked at change rather than absolute coverage levels. It is important to keep in mind that coverage was low for many of the indicators. Except for the immunization variables, where coverage tended to be high, it was much lower for many other potentially life-saving interventions. For example, the percentage of women reporting the use of a skilled birth attendant ranged from 6% to 83%, with a median value of 47% for the 27 countries; for appropriate treatment of diarrhea, values ranged from a low of 18% to a high of 75%, with a median of 37%. These data suggest that there is great room for improvement, even in countries that have already made substantial progress.

## Conclusions

Although this study is limited by its ecologic nature and the limited number of countries and information that were available for inclusion, we nonetheless feel that the method of examining average annual change rather than looking at absolute levels of outcomes should be considered in future studies examining the impact of change at a population level. More importantly, however, the findings on the apparent role of clinical services in further reducing under-five mortality may provide support to current efforts to improve the availability of care at community level and to make needed improvements in the health infrastructure, manpower, and logistic and supply systems.

## Abbreviations

AAC: Average annual change; DHS: Demographic and Health Surveys; GDP: Gross domestic product; iCCM: Integrated community case management; MDG: Millennium Development Goals; PCA: Principal component analysis; UNICEF: United Nations Children's Fund

## Competing interests

The authors declare that they have no competing interests.

## Authors' contributions

NB contributed to the conception, analysis, and writing of the article. MC contributed to the conceptual framework and writing. ASK performed the statistical analyses and contributed to the writing. DW contributed to the conception, developed the data-base, and provided input into the analysis and writing. All authors approved the final manuscript.

## Acknowledgements and funding

The authors wish to thank David Brown and Attila Hancioglu for their helpful comments and suggestions, to Mary Gray and David Olsen for their invaluable advice on data analysis, and Danielle Burke and Carlos Carrera for their assistance in identification of data bases used in these analyses. No specific funding was provided for this project, which was considered a routine UNICEF activity.

## Pre-publication history

The pre-publication history for this paper can be accessed here:

http://www.biomedcentral.com/1471-2458/11/456/prepub
